# Editorial: Targeting the microbiota to attenuate chronic inflammation

**DOI:** 10.3389/fimmu.2023.1202222

**Published:** 2023-04-18

**Authors:** Elena Moreno, Marius Trøseid, Ivan Vujkovic-Cvijin, Giulia Marchetti, Laura Martín-Pedraza, Sergio Serrano-Villar

**Affiliations:** ^1^ Department of Infectious Diseases, Hospital Universitario Ramón y Cajal, Facultad de Medicina, Universidad de Alcalá, Instituto Ramón y Cajal de Investigación Sanitaria (IRYCIS), Madrid, Spain; ^2^ Centro de Investigación Biomédica En Red (CIBER) de Enfermedades infecciosas (CIBERINFEC), Instituto de Salud Carlos III, Madrid, Spain; ^3^ Section for Clinical Immunology and Infectious Diseases, Oslo University Hospital Rikshospitalet, Oslo, Norway; ^4^ Department of Biomedical Sciences & F. Widjaja Inflammatory Bowel Disease Institute, Karsh Division of Gastroenterology and Hepatology, Cedars-Sinai Medical Center, Los Angeles, CA, United States; ^5^ Clinic of Infectious Diseases, Department of Health Sciences, University of Milan, ASST Santi Paolo e Carlo, Milan, Italy

**Keywords:** microbiota, inflammation, biomarkers, omics, personalized medicine (PM)

## Introduction

Although the whole human body is a complex ecosystem inhabited by very different microorganisms, including bacteria, fungi, archaea, and viruses, studies about gut bacteria have received the most attention. The relationship between the gut microbiota and the immune system has been the focus of intense research [recently reviewed at (1)]. Chronic inflammation correlates with an altered microbiota composition in the context of inflammatory bowel disease, colorectal cancer, frailty, metabolic endotoxemia, and non-communicable diseases (2–6). Specifically, many studies have addressed the role of different microbiome components on chronic inflammation (7, 8). For example, some microbes adhered to the intestinal epithelium can locally induce Th17 responses (9), while others can exert distant effects on different organ systems through metabolite production (10, 11). This has fueled research aimed at shaping these interactions by either redesigning the entire bacterial community or administering specific relevant bacterial strains that are presumably beneficial (12). However, most studies targeting the microbiota to elicit protective immune responses are only exploratory, with limited sample sizes and assessing multiple outcomes. Therefore, it is unclear how we can induce stable, beneficial changes in the gut microbiota.

This Research Topic highlights translational research and clinical trials evaluating the immunological effects of interventions on the gut microbiota, such as dietary or pharmacological interventions, probiotics, fungus, and other compounds. In addition, it focuses on specific mechanisms by which the microbiota can affect chronic inflammation in different diseases.

## Interventions on the gut microbiota in chronic inflammation diseases

This Research Topic addresses interventions on the gut microbiota from different perspectives.

According to new probiotic characterizations, Blázquez-Bondia et al. show the effect of a novel probiotic in a double-blind placebo-controlled clinical trial (RECOVER study). The i3.1 probiotic (a mixture of *L. plantarum* and *P. acidilactici* and a fiber-based prebiotic) improved immune reconstitution in people with HIV with impaired immunological recovery under stable antiretroviral therapy. There were no major adverse effects related to the intervention, and a slight increase in CD4/CD8 ratio as well as a decrease in pathways abundances were found in the active arm. Saghari et al. report the effects of three monoclonal microbial formulations of *L. lactis* spp. *cremoris* (EDP1066) on the immune response to a marine mollusk protein used to “mimic” an immune response in healthy volunteers. They assessed three different probiotic formulations to evaluate various exposure sites within the gastrointestinal tract. The immunomodulatory effect was assessed by quantifying circulating regulatory T cells and by stimulation of monocyte and lymphocyte with the Toll-like receptor 4 ligand lipopolysaccharide (LPS) and phytohemagglutinin (PHA), respectively. However, the results did not show a significant immune modulation measured as an antibody response to the challenge with the mollusk protein.

Plant compounds have mainly been used in the history of medicine. Liu et al. assessed the role of caesaldekarine, a cassane diterpenoid isolated from the plant *Caesalpinia bonduc*, to ameliorate colitis in mice. The mechanisms involved suppression of tissular inflammation, intestinal barrier integrity maintenance, and increased Lactobacillus abundance.

Fungi have been rarely been assessed as probiotics, with the exception of Saccharomyces Boulardii. Wang et al. evaluated the effects of dietary supplementation with *Tolypocladium sinense*, a mycelium isolated from a Chinese caterpillar, against obesity. This intervention affected the inflammatory response and oxidative stress levels by regulating lipid metabolism, such as decreasing short-chain fatty acid content. These results were further confirmed after fecal transplantation in mice.

Lastly, some traditional Chinese medicine products, as presented by Zhu et al., have been potentially related to improving different liver-related diseases through microbiota regulation mechanisms. Their review describes targeting microbiota studies to treat liver conditions, such as alcoholic disease, nonalcoholic disease, autoimmune disease, liver injury, and cancer.

## Advancing translational research in the microbiome field

Direct intervention studies are essential for determining the causal effects of the microbiome on disease pathogenesis. But it is also important to understand the mechanisms underlying specific microbiota actions in the host to design efficient and effective treatments. However, as described in Moreno et al., for the particular cases of HIV and HPV infection, most studies are based on highly dimensional datasets and address multiple outcomes, which hampers transferring the results to the clinic. The review also highlights the need for standardization of methods and encourages more hypothesis-driven studies.

Other studies in this Research Topic report novel mechanisms by which the microbiota can contribute to inflammation.

Ling et al. evaluated 140 school-aged children (6-12 years) from China (92 with depression and 48 healthy controls). They analyzed the correlations between gut microbiota profiles and host immune response measured as the expression of 27 cytokines. Patients with depression exhibited enrichment for proinflammatory genera (*Streptococcus*) and some inferred immunomodulatory metabolites (e.g., increase in membrane transport, signal transduction, and metabolism of other amino acids in children with depression), which correlated with increased levels of proinflammatory cytokines such as IL-17.

Fonseca et al. describe the anti-inflammatory and immunomodulatory properties of extracellular vesicles produced by the prominent human gut commensal bacterium *Bacteroides thetaiotaomicron*. By administering these vesicles to mice with colitis, they report important factors in anti-inflammatory and immunomodulatory responses, showing a reduction in intestinal inflammation, upregulation of the anti-inflammatory cytokine IL-10, and even epigenetic reprogramming.

As mentioned above, inflammation and HIV are two deeply interconnected factors. Littlefield et al. described the etiology of gastrointestinal inflammation among men who have sex with men and their link with gut microbiome composition. They found specific fecal soluble immune factors, such as calprotectin, a clinically relevant marker of gastrointestinal inflammation in men who have sex with men independently of their HIV status. They also observed differences in markers of bacterial translocation (elevated levels of plasma, sCD14, and sCD163) and in an *in vitro* system. These data indicate a connection between fecal soluble immune factors composition, decreased intestinal barrier function, and bacterial-induced systemic inflammation.

Additional mechanisms by which some therapeutic anti-inflammatory interventions could affect, and be affected by, the microbiota are addressed in this Research Topic.

Johnson et al. assess the effect of treatment with the humanized monoclonal antibody anti-α4β7 on microbiota composition. Vedolizumab administration to SIV-infected macaques led to different results than previous studies. The intervention elicited the maturation of macrophages associated with dysbiosis markers previously identified as predictors of HIV replication, immune activation, and changes in viral loads in tissues. This point towards a possible future modulation of gut immune functions to improve treatments for HIV infection.

Zhou et al. evaluate the involvment of the gut microbiota in the efficacy of the anti-rheumatic drug methotrexate. Patients with rheumatoid arthritis showed a decreased abundance of intestinal *Bacteroides fragilis* after methotrexate treatment. Transplantation of *Bacteroides fragilis* or supplementation with butyrate restored the methotrexate efficacy in collagen-induced arthritis mice pretreated with antibiotics.

Finally, four articles suggest a potential role of the microbiota in the activation of host pathways that have been linked to thepathogenesis of different conditions. First, Ancona et al. review the implications of gut dysbiosis in COVID-19 and long-COVID syndrome. They focus on the confounding factors in the previous literature and, more specifically, on studies of airway microbiota and long-COVID with neurological symptoms. Second, Omaru et al. reviewed the activation of NOD1/NOD2 receptors in chronic liver disease. This occurs through the regulation of proinflammatory cytokine responses leading to the development of chronic liver diseases, including hepatocellular carcinoma. Third, Fan et al. propose using the aryl hydrocarbon receptor as a therapeutic target for ischemic stroke by describing its role in the “microbiota-gut-brain axis” as a receptor of tryptophan metabolites that is impacted by gut microbiota. Finally, Li et al. review the impact of the oral microbiota on cardiometabolic health. Microbiota metabolites in the oral cavity, affected by oral dysbiosis, periodontal disease, and dental plaque, have been associated with cardiovascular disease occurrence. According to this, they discuss the potential of oral microbiota transplantation as a therapeutic intervention.

## Perspectives

This Research Topic includes studies that evaluate the potential of microbiota to attenuate chronic inflammation. Some of these studies assessed direct interventions on gut microbiota in different diseases and aimed to characterize the immunological effects. Other studies describe specific mechanisms underpinning these relationships, such as changes in metabolic routes or regulation of particular host factors related to immune responses. Finally, some of these studies review host-microbiota interactions in different conditions and suggest novel approaches to improve health

However, this field is still in its infancy, and more studies are required. We must unravel the specific mechanisms by which microbiota modulates the immune system. Such approaches could become helpful in improving outcomes in diseases characterized by chronic inflammation.

## Author contributions

EM drafted the manuscript; all authors contributed to the Research Topic, reviewed and accepted the last version of the manuscript.
